# Biomechanical properties of porcine meniscus as determined via AFM: Effect of region, compartment and anisotropy

**DOI:** 10.1371/journal.pone.0280616

**Published:** 2023-01-20

**Authors:** Kevin Orton, Wyndham Batchelor, Noel M. Ziebarth, Thomas M. Best, Francesco Travascio, Alicia R. Jackson

**Affiliations:** 1 Miller School of Medicine, University of Miami, Miami, Florida, United States of America; 2 Department of Biomedical Engineering, University of Miami, Coral Gables, Florida, United States of America; 3 Department of Orthopedics, University of Miami Sports Medicine Institute, Coral Gables, Florida, United States of America; 4 Department of Mechanical and Aerospace Engineering, University of Miami, Coral Gables, Florida, United States of America; 5 Max Biedermann Institute for Biomechanics at Mount Sinai Medical Center, Miami Beach, Florida, United States of America; Drexel University, UNITED STATES

## Abstract

The meniscus is a fibrocartilaginous tissue that plays an essential role in load transmission, lubrication, and stabilization of the knee. Loss of meniscus function, through degeneration or trauma, can lead to osteoarthritis in the underlying articular cartilage. To perform its crucial function, the meniscus extracellular matrix has a particular organization, including collagen fiber bundles running circumferentially, allowing the tissue to withstand tensile hoop stresses developed during axial loading. Given its critical role in preserving the health of the knee, better understanding structure-function relations of the biomechanical properties of the meniscus is critical. The main objective of this study was to measure the compressive modulus of porcine meniscus using Atomic Force Microscopy (AFM); the effects of three key factors were investigated: direction (axial, circumferential), compartment (medial, lateral) and region (inner, outer). Porcine menisci were prepared in 8 groups (= 2 directions x 2 compartments x 2 regions) with n = 9 per group. A custom AFM was used to obtain force-indentation curves, which were then curve-fit with the Hertz model to determine the tissue’s compressive modulus. The compressive modulus ranged from 0.75 to 4.00 MPa across the 8 groups, with an averaged value of 2.04±0.86MPa. Only direction had a significant effect on meniscus compressive modulus (circumferential > axial, p = 0.024), in agreement with earlier studies demonstrating that mechanical properties in the tissue are anisotropic. This behavior is likely the result of the particular collagen fiber arrangement in the tissue and plays a key role in load transmission capability. This study provides important information on the micromechanical properties of the meniscus, which is crucial for understanding tissue pathophysiology, as well as for developing novel treatments for tissue repair.

## Introduction

Knee osteoarthritis (OA) is the most common form of OA affecting approximately 240 million people worldwide [1]. Associations have been found between damage to the meniscus and subsequent development of OA resulting from the loss of load distribution capability throughout the knee [2]. Meniscus lesions with an average annual incidence of 66 per 100,000 are the most common surgical procedure performed by orthopedic surgeons in the United States [3–5]. Meniscus lesions can be classified based on region, thickness, stability, and the most useful in guiding treatment decisions, tear pattern [6–8]. Tear pattern encompasses numerous considerations with regards to the region and orientation of the tear [9]. Continued research to understand the mechanical properties and how they vary throughout the meniscus is essential to understand the tissue’s structure- function relationship which may help guide the development of new treatment options.

The meniscus is a C-shaped piece of fibrocartilage that is situated between the femoral condyle and the tibial plateau (one in the lateral compartment and one in the medial compartment of the knee). These structures serve to increase the contact area of the joint through its entire range of motion which is essential in decreasing contact stresses throughout the joint. As the knee bears load, compressive forces are distributed over the contact area resulting in contact stresses inversely proportional to the contact area [10]. Since the meniscus increases the contact area, it also functions to decrease contact stresses on articulating surfaces. Studies have demonstrated this important function and have influenced the current trend in meniscus repair focusing on preservation of the tissue wherever possible [11]. Additional studies are necessary however to better understand the tissue’s microscopic biomechanical properties as a motivation to developing treatment approaches emphasizing restoration of structure and function.

**Fig 1:**
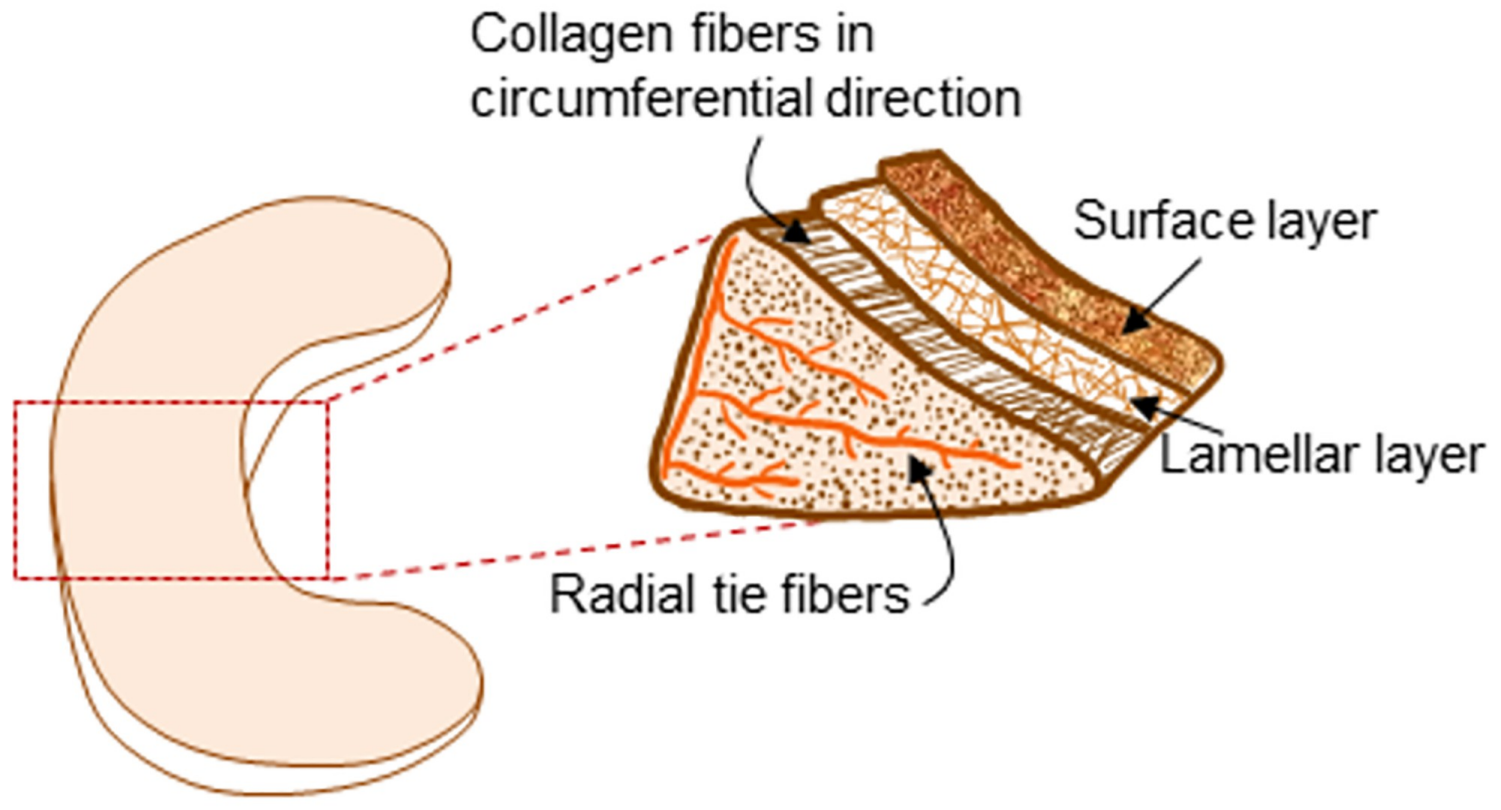
Schematic illustrating the basic collagen organization in the knee meniscus. The collagen fibers are arranged in parallel fiber bundles in the core region of the tissue, whereas less alignment is seen in the surface and lamellar zones.

Microscopically, the meniscus consists of a particular fiber orientation that has been described in detail, see Fig 1 [12–17]. On the surface of the meniscus, the fibers are randomly oriented. Below the surface, there are circumferential fiber bundles along with radial tie fibers. One study found that the radial tie fibers were more highly concentrated in the middle zone of the meniscus and on the exposed surfaces [14]. Another study identified structurally distinct regions of the menisci: an inner two-thirds and an outer one-third [13]. In the outer portion of the tissue (where the thickness or height is greater), the fibers are highly organized and primarily circumferentially oriented. In the inner portion of the tissue, the organization of the fibers are more randomly distributed, often radially orientated and parallel to the articular surface [12]. This particular fiber orientation is thought to be responsible for the meniscus’ ability to withstand high loads within the knee. The wedge shape of the meniscus functions to convert compressive loads into hoop stresses parallel with the circumferential fibers. These fibers, which are further organized into bundles, enable a series of shear forces to develop between them serving to counter radial deformation [18–20]. The force conversion and partial resistance to radial deformation provides understanding of the meniscus’s dual function in load bearing and joint stability, respectively. Furthermore, this particular fiber arrangement lends to anisotropic mechanical behavior of the tissue [20–32].

Atomic Force Microscopy (AFM) is a state-of-the-art nanotechnology originally designed for imaging surface topography. More recently, AFM has been developed for nanoindentation testing of cells and biological soft tissues [33]. It is a convenient technique that allows for investigating regional differences in local indentation properties of tissues. Recently, several studies have employed an AFM technique to investigate the mechanical properties of meniscus from varies species. The effects of region [24,34], compartment [35], direction [24,36], age [36], and degeneration [37] on the indention properties of the meniscus have been investigated. However, to our knowledge, no single study has investigated the effects of anisotropy and tissue inhomogeneity (medial vs. lateral and inner vs. outer) in hydrated meniscus.

In order to fill this gap in knowledge in the literature, the aim of this study was to investigate the compressive modulus in meniscus tissue and determine the effects of compartment, orientation, and region. Given the unique structural organization of the tissue extracellular matrix, we hypothesized that the compressive modulus is (1) anisotropic and (2) inhomogeneous in porcine meniscus. To test these hypotheses, we employed an AFM technique to measure the compressive modulus in porcine meniscus in different compartments (medial and lateral), different directions/tissue orientations (axial and circumferential), and different regions (inner and outer) in the tissue. The use of AFM allows the modulus to be measured at the nanoscale and within a hydrated and thus physiologically comparable environment.

## Methods

### Specimen preparation

**Fig 2:**
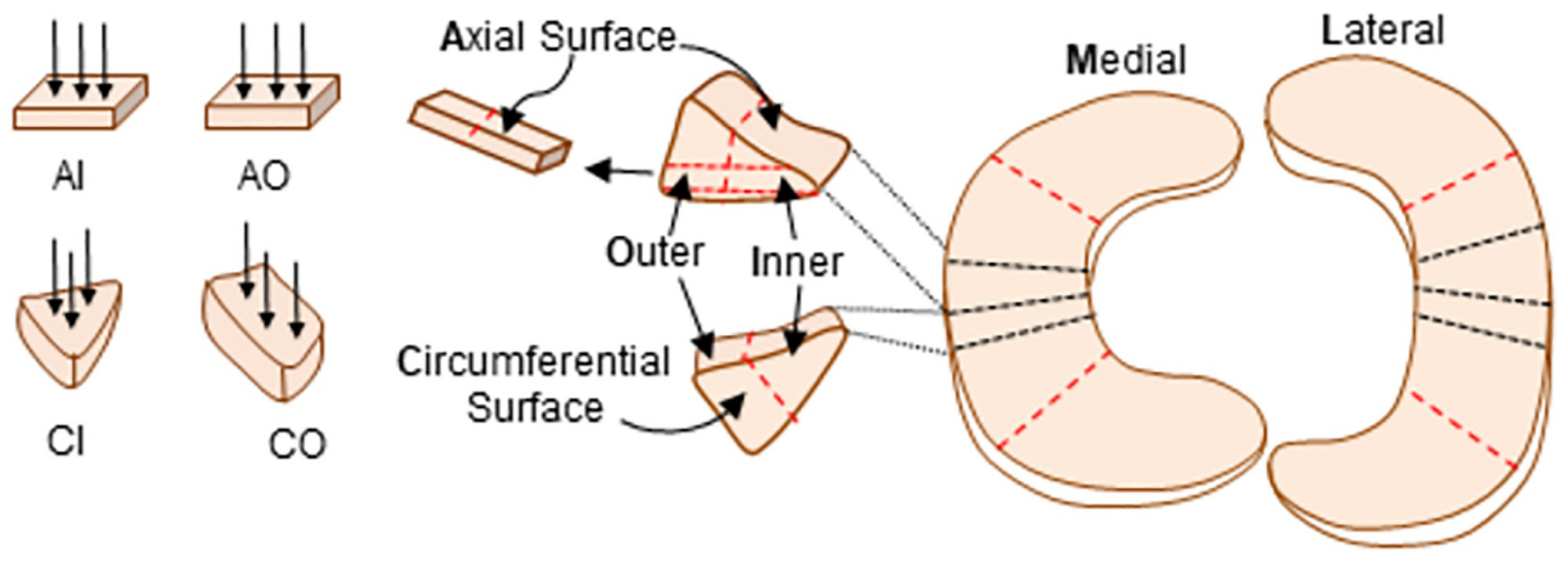
Orientations and locations of test specimens for eight groups investigated. Circumferential specimens had collagen fiber bundles running perpendicular to the testing surface, while axial specimens had bundles running across the testing surface. Outer zone included the outer third of the tissue, while inner was the remaining inner two-thirds. All specimens were taken from the central region of both medial and lateral porcine menisci, within the deep/core portion of the tissue. Eight groups were investigated based on compartment (medial (M) and lateral (L)), direction (axial (A) and circumferential (C)), and region (inner (I) and outer (O)).

Frozen porcine meniscus was obtained from a commercial tissue source (Animal Technologies, Inc., Tyler, TX) and stored in -20°C until the day of measurements; a total of 9 medial and 9 lateral menisci (age: 10–12 months) were used in this study. On the day of the experiment, the tissue was thawed, and two slabs of tissue were excised from the central region of the meniscus: one slab oriented in the circumferential direction, and one oriented in the axial direction, see Fig 2. A compresstome (VF-200-0Z, Precisionary, Natick, MA) was used to obtain a slice of the tissue that was uniformly 1.5 mm in thickness. The tissue was secured to the compresstome components in specific orientations in order to expose the correct surface for indentation once the cut was completed–one to expose the axial surface and the other to expose the circumferential surface. Each of the slices were then cut using a scalpel to result in one sample consisting of the inner two-thirds of the 1.5 mm tissue slice and one sample consisting of the outer one-third of the 1.5 mm slice (Fig 2). This provided four groups per meniscus for analysis: Axial Inner (AI), Axial Outer (AO), Circumferential Inner (CI), Circumferential Outer (CO). In addition, menisci were divided into medial and lateral groups, giving a total of 8 groups for analysis (medial axial inner-MAI, lateral axial outer-LAO, etc). For each group, nine samples were obtained.

### Mechanical testing

**Fig 3:**
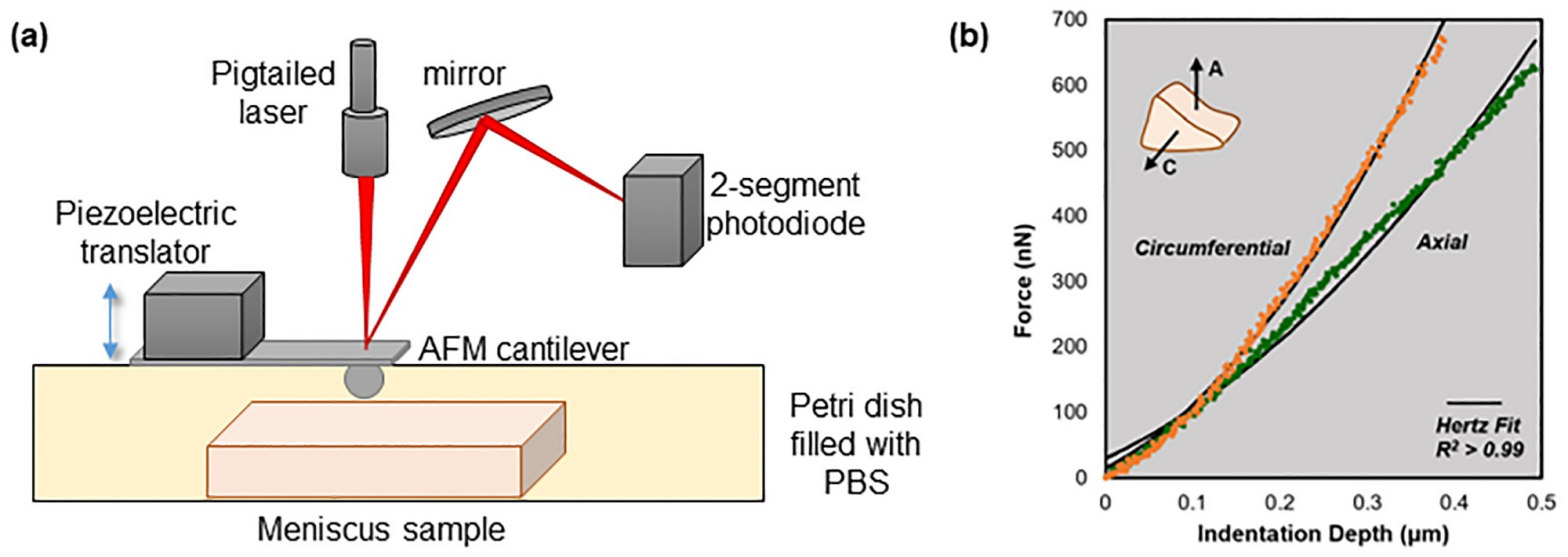
(a) Schematic showing basic setup of custom AFM. The cantilever is moved vertically with a piezoelectric translator that responds to a voltage applied. The cantilever is lowered onto the meniscus tissue specimen in the petri dish below. The cantilever bends, causing the beam from the laser diode to be deflected. The deflections are monitored by the photodiode. Data from cantilever deflections are then recorded for post processing to obtain the compressive modulus of the sample. (b) Representative force-indentation curves used for curve-fitting to determine compressive modulus. Curves shown are both outer samples from the same medial meniscus specimen.

Each sample was placed in a Petri dish and secured using super glue to prevent movement of the tissue during measurements. Caution was used to ensure that no glue was on the indentation surface. After the glue had completely dried, the sample was submerged in PBS with protease inhibitors (Roche) to prevent the breakdown of the tissue during testing. The Petri dish with the mounted sample was placed into a custom AFM designed for biomechanical measurements of cells and tissues, see schematic in Fig 3a [38–40]. All experiments were performed with both the sample and the cantilever submerged in PBS throughout the entire scan. The measurement location was controlled by moving the sample in the x-y plane (parallel to the anterior surface of the sample) using manual actuators. Once over the target location on the sample surface, the cantilever (5μm diameter borosilicate glass particle, 5.4 N/m) was used to indent the sample via a piezoelectric actuator that moves the cantilever vertically, perpendicular to the sample surface, with nanometer precision. In response, the cantilever undergoes a combination of indentation and deflection (bending) dependent on the softness of the sample. The bending of the cantilever is proportional to the force that the AFM probe tip exerts on the sample. The degree of bending is tracked with a laser and position-sensitive photodiode. The measurements were conducted using a cantilever approach and retraction speed of 15μm/s and a maximal indentation force of 1000V (resulting in <1μm indentation and <1μN force in all measurements). The recorded cantilever deflection-indentation curves were then used to derive the force-indentation curves for each measurement after factoring out the deflection of the cantilever on a hard surface; representative curves are shown in Fig 3b. The compressive modulus was calculated from the force-indentation curves using the Hertz model of spherical indenters [41]:

$$F=\frac{4E\sqrt{R}}{3(1-\nu^{2})}D^{3/2}$$

where *F* is the measured force in N, *E* is the compressive modulus in Pa, *R* is the cantilever tip’s radius (2.5μm), *ν* is Poisson’s Ratio for the sample (0.3[42]), and *D* is the indentation measured by the system.

Samples were each indented at least 10 times in each of three different locations on the sample. That is, at least ten measurements were taken in a given x-y location, then the sample was repositioned, moving it approximately 2 mm in both the x and y directions, and the process was repeated. A total of 3 locations were measured in each sample. AFM data was analyzed with a custom code in MATLAB to obtain the compressive modulus for each sample [38].

### Statistical analysis

Raw data was analyzed using the interquartile range method to identify and exclude outliers, as in our earlier study [43]. Using this method, any value 1.5 times above the third quartile or 1.5 times below the first quartile were excluded. Since at least 10 repeat measures were acquired per measurement location, we applied this outlier analysis method to the repeat measures for each location. If a point was determined to be an outlier in accordance with the interquartile range method, it was excluded from the average value for that location. Finally, the same process was applied to the average values for all three locations for each group, and any outliers were excluded from the averages for each sample. Based on this approach, only 2 measurement locations were excluded as outliers.

Statistical analyses were carried out using Minitab software (Minitab LLC, State College, PA). A total of 9 medial and 9 lateral menisci were obtained; from each sample, 4 specimens were prepared representing AI, AO, CI and CO groups. For each specimen, data from repeated measurements were averaged, to give a total of nine compressive modulus values (n = 9) for each of the 8 groups investigated, with each averaged modulus value coming from a distinct meniscus. A three-way ANOVA was used to investigate significant interactions and main effects of compartment, region, and direction on the measured compressive modulus across the eight groups investigated. A Fisher LSD test was used for post hoc comparisons to determine significant differences between groups. For all tests, the statistical significance was set to α = 0.05. When no statistical effect was found for a given factor, samples were pooled.

## Results

**Fig 4:**
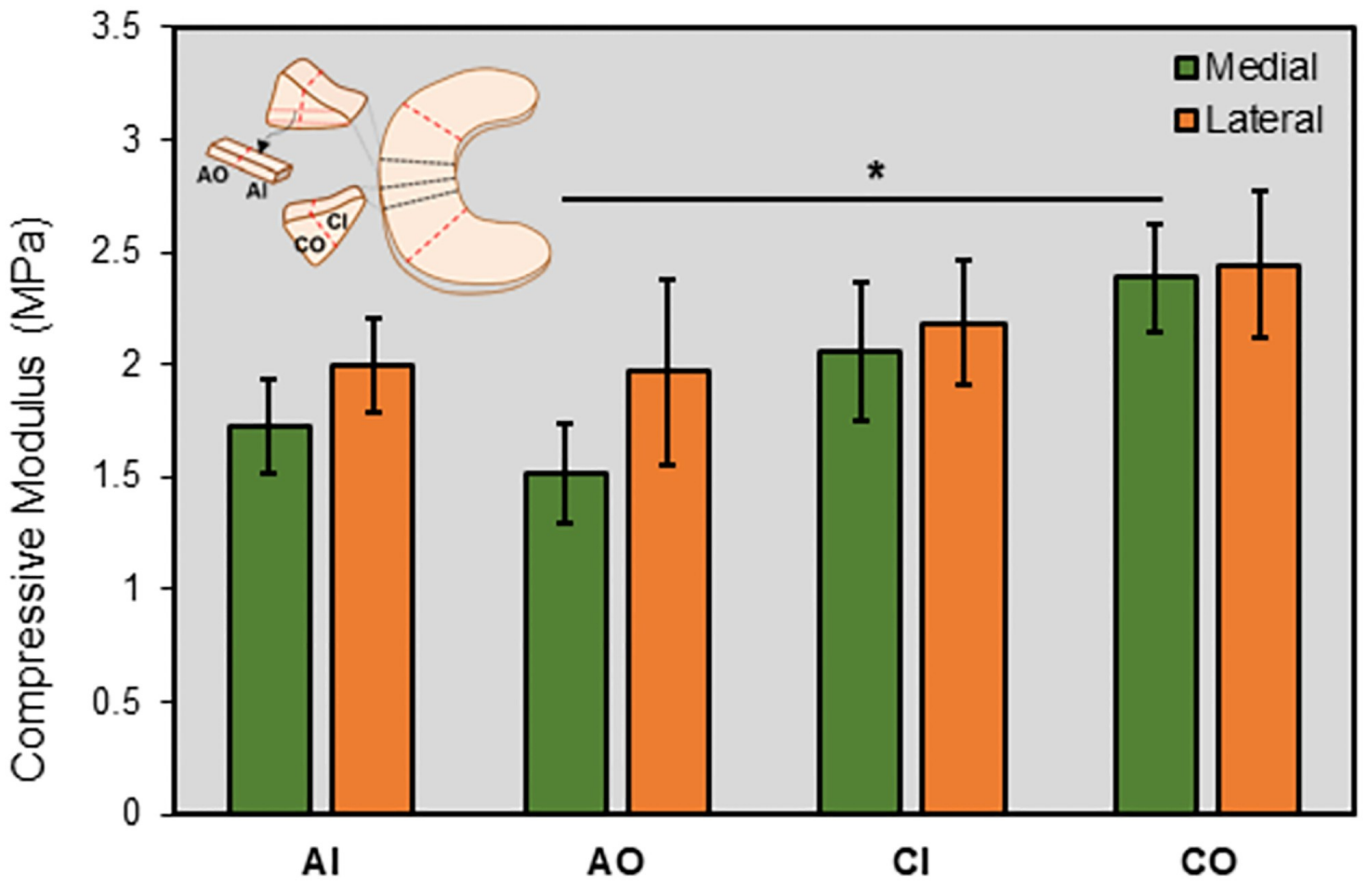
Results for compressive modulus of porcine meniscus for all eight groups investigated. For each group, n = 9. Data are shown as mean ± standard error of the mean (mean±SEM). Statistical significance is shown as *: p<0.05.

**Fig 5:**
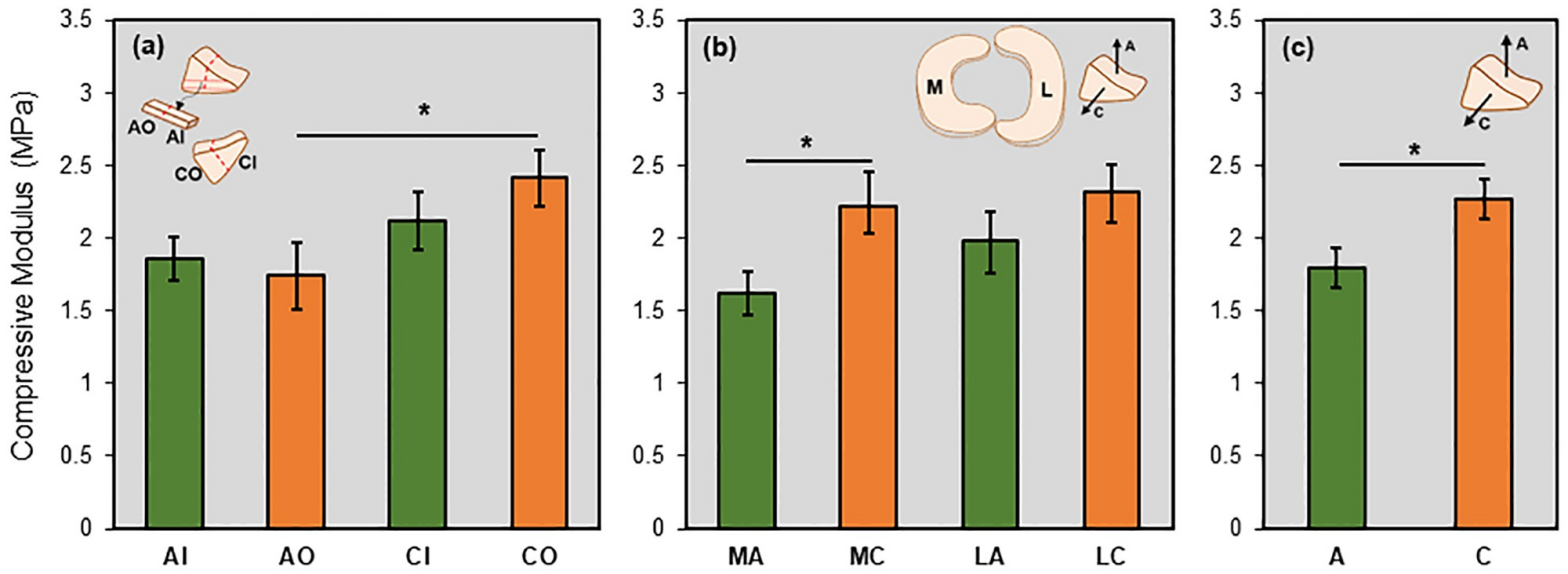
Results for compressive modulus of porcine meniscus for pooled samples. (a) Samples for medial and lateral groups are pooled; for each group, n = 18. (b) Samples for inner and outer groups are pooled; for each group, n = 18. (c) Samples for both compartments and regions are pooled; for each group, n = 36. Data are shown as mean ± standard error of the mean (mean±SEM). Statistical significance is shown as *: p<0.05.

The elastic modulus of the tissue ranged from 0.75 to 4.00 MPa for all groups investigated (Fig 4), with an averaged value across all groups of 2.04 ± 0.86 MPa (mean ± standard deviation). When divided into eight groups based on meniscus compartment (medial vs. lateral), region (inner vs. outer), and direction (axial vs. circumferential), a three-way ANOVA found that only direction had a significant effect on the compressive modulus (p = 0.024). There was no significant interaction found among factors. In post-hoc analysis, only MAO and MCO groups were different (MCO > MAO, p = 0.035). In general, the values for circumferential groups were higher than the axial group for the same region and compartment. When pooling data from the medial and lateral compartments, we found only AO and CO groups were different (CO > AO, p = 0.024). When pooling data from inner and outer regions, we found only MC and MA groups were different (MC > MA, p = 0.042). Therefore, due to lack of statistically significant differences for the effect of compartment and region, we compared the results for modulus for pooled data in the axial and circumferential direction and found that the modulus in the circumferentially oriented specimens was higher than that in the axially oriented specimens (p = 0.024), indicating anisotropic micromechanical properties are present in the tissue. These comparisons for pooled samples are shown in Fig 5.

## Discussion

Knowledge of compressive properties of the meniscus is crucial to understanding structure-function relations in the tissue, given that the meniscus serves to convert vertical compressive loading to tensile circumferential and shear loading during normal daily activities. In this study, the effects of region, direction, and compartment of the meniscus on tissue compressive modulus were investigated using AFM. To our knowledge, this is the first study to investigate the effects of these three factors in a single study. Overall, we found significant anisotropic behavior of the compressive modulus in porcine meniscus, but no differences related to the region or compartment of the tissue. Understanding how mechanical properties of the meniscus vary for different locations and orientations is critical for developing effective tissue replacement and/or regeneration strategies.

Our findings indicate that there is a significant anisotropic trend for meniscus tissue compressive modulus, being higher in the circumferential direction than in the axial direction. This behavior was expected due to the particular organization in the meniscus extracellular matrix. In the core portion of the tissue, collagen fiber bundles run in the circumferential direction and are highly organized in a parallel arrangement [12,18]. As noted, this structural organization allows the tissue to withstand hoop stresses that arise when the meniscus is compressed axially, as during walking or other daily activities. Our findings are in agreement with an earlier AFM study by Li et al (2017) that found that the modulus was higher parallel to the collagen fiber bundles (i.e., in the circumferential direction) compared to orthogonal to the fibers [24]. Furthermore, several previous studies have found that other mechanical properties of meniscus tissues are anisotropic [20–32]. A previous study using unconfined compression testing have found that the meniscus has the highest compressive modulus in the axial direction [28]. However, it has been suggested that this difference may be due to the magnitude of strain applied during nanoindentation testing, which results in fiber uncrimping and sliding rather than tensile stretching [24].

Interestingly, our results show that, when dividing specimens into medial and lateral groups, only the medial groups showed statistically significant anisotropic trends (e.g., MCO > MAO, p = 0.035; MC > MA, p = 0.042). A recent study by Gonzalez-Leon et al. (2022) found a higher anisotropic index in the medial meniscus compared to the lateral meniscus in the minipig model, which is in agreement with our findings [32]. This is likely owing to the lower compressive modulus in the axial direction (orthogonal to the collagen fiber direction) in medial samples. Similarly, that same study also found reduced mechanical properties orthogonal to the fibers (in their study, in the radial direction) for medial menisci. More investigations are needed to further understand any differences in anisotropic trends that may exist between medial and lateral tissues, including both mechanical and compositional studies. Such information could be beneficial for elucidating meniscus pathophysiology, as well as developing site specific tissue substitutes that mimic native tissue.

We did not find a significant effect of compartment (i.e., medial vs. lateral) on the compressive modulus. This is in agreement with an earlier AFM study by Li et al (2015) that found no difference in modulus for murine medial and lateral menisci [35]. Moreover, previous studies measuring mechanical properties of the tissue employing other methods have also produced mixed findings as to the effect of tissue compartment on meniscus mechanics [32,44–47]. It is noteworthy that the trends shown in Figs 4 and 5 indicate that the compressive modulus is in general higher in the lateral meniscus than in the medial meniscus for all groups, although this did not reach significance (p = 0.275). Previous studies have shown that the lateral meniscus bears higher loads, is more mobile, and less likely to tear than its medial counterpart [19]. The higher modulus would allow the lateral meniscus to withstand the larger loads seen physiologically.

When comparing the inner two thirds of the tissue with the outer one third, we did not find any significant differences in the modulus between comparable groups. Some previous AFM studies found no significant effect of region [24,35], while others did find differences [34,37]. Of note, in the present study for both the medial and lateral groups, the modulus in the inner axial group was, in general, higher than in the outer axial group, whereas the opposite was true for the circumferential groups. This behavior is likely related to the difference in organization of the matrix in the inner and outer regions of the tissue. The inner meniscus withstands compressive forces in the axial direction during normal loading of the knee joint, whereas the outer portion of the meniscus is subjected to tensile forces (along the circumferential direction). This function is reflected in the organization of the tissue matrix: a previous study by Andrews et al showed that in the outer one third of the meniscus, there is a high degree of organization, with collagen fibers arranged parallel to one another [12]. On the other hand, in the inner one third of the tissue, the same study found a much more random arrangement of fibers. In agreement with this study, our results showed a significant anisotropic trend in the outer section of the tissue, but there was no statistically significant difference (p = 0.376) in the inner portion.

Our results for compressive modulus of the tissue are within the range of those in the literature [24,25,34–36,46–49]. Deviations between values in the literature and those reported in the current study are likely the result of differences in measurement techniques and analysis, specimen preparation, and species used. For instance, previous AFM studies have generally used very thin (~10–20 μm) specimens [24,35–37], in comparison to this study which utilized 1.5mm thick specimens. In particular, our findings are in strongest agreement with previous AFM and nanoindentation studies that used fully hydrated and thicker meniscus samples [46,48–50]. Other AFM studies have used a value of Poisson’s ratio ranging from 0 to 0.04 [24,34–36], while we used ν = 0.3 based on a recent study directly measuring the ratio in meniscus tissue [42]. However, similarly to that noted in Li et al (2015) [35], varying the value of ν from 0 to 0.49 only results in a ~30% variation in values, and no change in the statistical findings of the study.

There are a few limitations of this study that should be noted. First, we chose to investigate the behavior of the core portion of the meniscus, which allowed us to investigate the degree of anisotropy in the tissue. However, as shown in Fig 1, the meniscus is highly organized, and contains an outer, superficial layer that has a more random organization; this layer is in contact with the femoral and tibial surfaces in the knee [17]. Previous studies have shown differences in mechanical properties between the surface and interior region of the tissue [24,31,51,52]. This deserves further investigation to better understand the structure-function relations and heterogeneity in the tissue. In addition, we also used porcine meniscus tissues in this study, whereas human tissues may have a higher clinical relevance. However, the porcine meniscus is often used to model the human tissue due to similarities in shape, composition, and properties [45,53–56]. Finally, this study in porcine meniscus was limited to a relatively small sample size (n = 9). In the future, we will investigate these properties in human tissues with a larger sample size, in order to more fully understand the particular trends and structure-function relations in the tissue.

In summary, we investigated the effect of three factors on the compressive modulus of porcine meniscus: region (inner vs. outer), compartment (medial vs. lateral), and direction (axial vs. circumferential). Our findings indicate that there is significant anisotropy in the mechanical behavior of the meniscus in compression, but no significant effect of other factors or interactions among factors was observed. Despite limitations, this study provides important quantitative information about the structure-function relations of the meniscus. Such information can be harnessed to not only aid in better understanding the pathophysiology of the meniscus and related osteoarthritis, but also for the development of novel treatment strategies to restore and/or regenerate the tissue.

## Supporting information

S1 Data (XLSX)
